# SELEX-Based Aptamer Technologies for Toxin Analysis: Screening, Optimization, and Computational Assisted Design

**DOI:** 10.3390/toxins18070293

**Published:** 2026-07-03

**Authors:** Xinrui Shang, Chengming Yang, Huiyun Deng, Lianghua Wang, Mingjuan Sun

**Affiliations:** 1 Department of Student Team, College of Basic Medical Sciences, Naval Medical University, Shanghai 200433, China; xrshang0701@163.com (X.S.); cmyang0903@163.com (C.Y.); hydeng919@163.com (H.D.); 2 Department of Biochemistry and Molecular Biology, College of Basic Medical Sciences, Naval Medical University, Shanghai 200433, China; 3 Key Laboratory of Biosafety Defense (Naval Medical University), Ministry of Education, Shanghai 200433, China

**Keywords:** toxin detection, biosensor, nucleic acid aptamer, SELEX technology, aptamer optimization, computational design, aptasensor

## Abstract

The accurate and sensitive detection of toxin contamination remains a pressing challenge for food safety, environmental integrity, and public health, because conventional analytical methods suffer from high costs, poor field stability, and inadequate sensitivity for trace-level emerging contaminants. In this review, we provide a comprehensive overview of biosensor technologies for toxin detection, with a dedicated focus on nucleic acid aptamers and SELEX (Systematic Evolution of Ligands by Exponential Enrichment) technology. We systematically categorize nine SELEX variants developed for toxin detection, covering target-immobilized, library-immobilized, non-immobilized, cell-based, and high-throughput platforms, with an emphasis on their selection principles, applicability, and limitations. This review discusses computationally assisted aptamer discovery (e.g., AI-based sequence generation and molecular docking) as well as experimental post-SELEX optimization strategies such as cyclization, multivalent assembly, and structure-switching design. We then discuss key challenges and future perspectives, highlighting the shift from method-oriented to demand-oriented aptamer development through integrated SELEX strategies and AI-assisted design. Overall, this review covers mainstream SELEX technologies, aptamer selection, computational design, experimental optimization, and sensor integration to serve as a reference for next-generation toxin detection applications.

## 1. Introduction

Toxin contamination poses a pervasive global threat to food safety, environmental integrity, and human health, with its presence across foodstuffs, drinking water, soil, and air matrices [1,2,3]. Notably, a large proportion of these toxins are categorized as Emerging Contaminants (ECs)—naturally occurring toxicants that have recently garnered widespread attention owing to their unclear environmental fate, insufficient monitoring protocols, and potent toxicological effects even at trace levels, including mycotoxins, freshwater/marine algal toxins (e.g., microcystins) [4,5], novel pesticide toxins [6], and bacterial exotoxins from antibiotic-resistant strains [7,8]. Unlike conventional pollutants, these EC-associated toxins often evade routine environmental and food safety monitoring, yet their trace-level accumulation can trigger severe toxicological effects (e.g., carcinogenicity, neurotoxicity, and hepatotoxicity) [9,10,11,12], thereby imposing enormous public health risks and economic burdens worldwide. Thus, the accurate, sensitive, and rapid detection of toxins—particularly ECs—is critical for early hazard identification, timely risk mitigation, and effective protection of human health and ecological security [13].

Toxin detection methods can be broadly classified into analytical and non-analytical approaches. Well-established analytical methods are dominated by HPLC- and LC-based techniques [5,14,15,16]; while HPLC-FLD/UVD and LC-MS enable unambiguous qualitative and quantitative analysis of most toxin groups, their widespread application is severely constrained by the overreliance on certified reference standards, exorbitant instrumentation costs, labor-intensive and time-consuming sample pretreatment, and the need for professional operational expertise. These limitations confine such techniques to laboratory-based analysis rather than point-of-site testing (POST) [17]. Non-analytical methods include mouse bioassays, cell-based assays, immunoassays, enzyme inhibition assays, receptor binding assays, and biosensors [17,18,19,20,21,22]. Among these, enzyme-linked immunosorbent assay (ELISA) and lateral flow assay (LFA) test strips alleviate the reliance on certified reference standards but exhibit critical inherent limitations: they cannot discriminate between toxin analogs and display insufficient sensitivity for trace-level small-molecule toxins. Furthermore, antibodies possess poor field stability and are difficult to generate for small-molecule toxins with limited epitopes, while enzymes are susceptible to denaturation and activity loss under non-laboratory conditions—these two issues severely compromise the reproducibility and reliability of detection results [23,24,25]. Collectively, these drawbacks constitute a critical technological bottleneck for toxin detection, generating an urgent demand for a novel detection platform that integrates high sensitivity, excellent stability, low cost, on-site operability, and a broad detection range.

In this context, biosensor technologies have emerged as a promising alternative to conventional analytical and non-analytical detection methods, and have become a research hotspot for EC-associated toxin detection in food safety and environmental monitoring fields [26,27]. A biosensor is a bio-analytical device that integrates a bio-recognition element with a transducer, which converts specific biological recognition responses into measurable electrical signals [28]. For toxin detection, biosensors are classified by their bio-recognition elements into several types: enzymatic, immunosensor (antibody-based), receptor-based, tissue/organelle/cell-based, and nucleic acid-based biosensors [29,30,31,32]. Enzymatic and immunosensors are the most mature traditional biosensor types, while receptor and tissue/organelle-based biosensors have advanced rapidly in the field of marine toxin detection. However, these traditional biosensors are severely limited by inherent defects of their bio-recognition elements: enzyme denaturation in harsh field conditions, poor antibody stability and preparation difficulties for small-molecule toxins, unstable natural targets for receptor-based sensors, and high professional demands for cell/tissue culture for tissue/organelle-based sensors. Collectively, these limitations mirror the shortcomings of conventional analytical and non-analytical methods—high cost, poor field stability, and insufficient sensitivity—underscoring the need for a new class of biorecognition elements that can overcome these barriers.

In contrast to these bio-recognition elements, nucleic acid aptamers offer a combination of chemical stability, synthetic accessibility, and broad target scope. To overcome these critical limitations, nucleic acid aptamers have garnered extensive attention in recent years as novel high-performance biorecognition elements, and SELEX (Systematic Evolution of Ligands by Exponential Enrichment) technology—the core in vitro aptamer screening method—has become a key enabling technology for developing high-performance toxin-detecting biosensors [33]. As artificial single-stranded DNA/RNA (ssDNA/RNA) molecules with high affinity and specificity for target analytes, aptamers outperform traditional enzymes, antibodies, and natural receptors for toxin detection applications: they allow for reproducible chemical synthesis and modification, retain excellent structural and functional stability under extreme conditions (e.g., high temperature, extreme pH), and can recognize a broad range of EC-associated toxins, including small-molecule mycotoxins and macromolecular bacterial exotoxins. Iterative optimization of SELEX technology and its derivative platforms has significantly improved the screening efficiency of high-affinity aptamers against diverse toxins. Meanwhile, the integration of computational simulation tools, AI-assisted design, and experimental validation has advanced the development of a multi-scale aptamer optimization framework, thus laying a solid foundation for the fabrication of ultrasensitive and anti-interfering aptamer-based biosensors (aptasensors).

**Figure 1:**
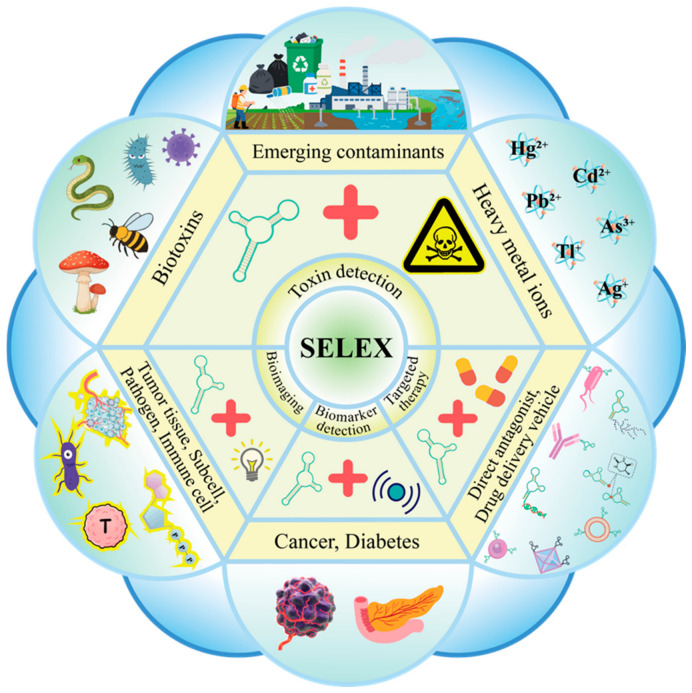
Overview of SELEX application domains. Centered on SELEX technology, this diagram illustrates multiple research branches of aptamer utilization. The toxin detection module includes biotoxins, toxic heavy metal ions, and industrial and agricultural emerging environmental pollutants. Additional application fields cover disease biomarker bioimaging, targeted therapeutic delivery, and specific identification of pathogens, tumor tissues and immune cells.

As illustrated in Figure 1, SELEX technology has been successfully applied to diverse fields including pathogen detection, tumor targeting, biomarker discovery, and targeted therapy. This review focuses specifically on its application to toxin detection, with an emphasis on emerging contaminants and biotoxins.

Despite these advances, several critical gaps remain. First, although various SELEX variants have been developed for toxin detection, a systematic categorization of these platforms—with emphasis on their selection principles, applicability across diverse toxin types, and inherent limitations—is still lacking. Second, the integration of computational simulation, AI-assisted design, and experimental validation into a unified aptamer optimization framework has not been comprehensively reviewed for toxin detection applications.

Based on this research gap, this review comprehensively summarizes the recent advances in biosensor technologies for toxin detection, with a dedicated focus on nucleic acid aptamers and SELEX technology optimization. We first systematically categorize nine SELEX variants developed for toxin detection with an emphasis on their selection principles, applicability across diverse toxin types, and inherent advantages and limitations. Next, we elaborate on the multi-scale aptamer optimization framework integrating computational simulation tools and experimental validation approaches (truncation, chemical modification, multivalent aptamer assembly). Finally, we discuss key challenges and future perspectives, highlighting the shift from method-oriented to demand-oriented aptamer development through integrated SELEX strategies and AI-assisted design.

## 2. Basic Principles of SELEX

**Figure 2:**
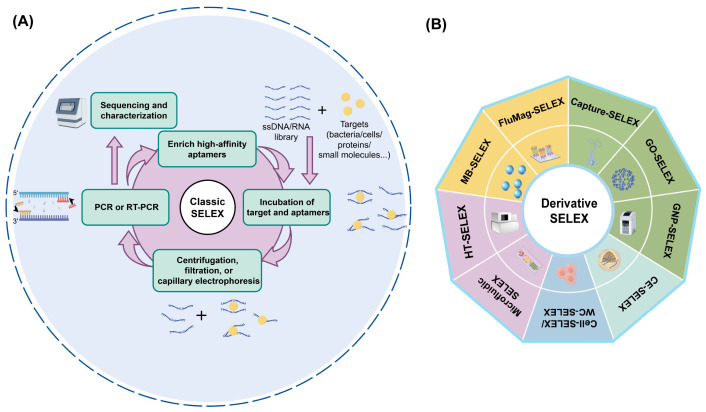
Schematic illustration of classic SELEX workflow and derivative SELEX variants for toxin detection. (**A**) Schematic of the iterative core workflow of classic SELEX, including incubation, separation, amplification, and enrichment. The curved arrows denote the cyclic execution sequence of each screening step; the small directional arrows in the PCR subgraph indicate the 5′ to 3′ polarity of single-stranded nucleic acid strands. (**B**) Classification of nine representative derivative SELEX variants organized into five major categories. (Some drawing elements are from www.figdraw.com).

Aptamers are short synthetic ribonucleic acid (RNA) and deoxyribonucleic acid (DNA) molecules that were first reported independently by Ellington et al., Tuerk et al. and Robertson et al. in the early 1990s [34,35,36]. SELEX is an in vitro selection technology that isolates high-affinity nucleic acid aptamers from large random sequence libraries through iterative rounds of incubation, partitioning, and amplification. Notably, almost all aptamers generated via SELEX exhibit strong binding affinity to their targets, with a dissociation constant (Kd) typically ranging from the nanomolar to picomolar scale [37]. The classic SELEX workflow involves: (1) incubation of the ssDNA/RNA library with the target toxin; (2) separation of toxin-aptamer complexes from unbound sequences (e.g., via centrifugation, filtration, or capillary electrophoresis); (3) amplification of bound sequences using PCR (for DNA) or RT-PCR (for RNA); and (4) repetition of these steps (3–10 rounds) to enrich high-affinity aptamers, followed by sequencing and characterization (Figure 2A). Classical SELEX experiments generally employ initial random oligonucleotide libraries containing 10^13^ to 10^15^ unique sequences, where each oligonucleotide consists of a variable random core of 20–80 nucleotides (with 30–50 nucleotides as the most widely adopted length) flanked by 18–21-nucleotide conserved primer-binding regions at both the 5′ and 3′ termini [38]. To improve specificity against closely related toxin analogs, counter-selection steps (e.g., pre-incubation with structurally similar non-target molecules) can be incorporated into the selection scheme, typically after the initial enrichment rounds. Moreover, to overcome the limitations of classic SELEX, derivative SELEX methods have emerged for diverse applications (Figure 2B), with inherent strengths in toxin detection: addressing small-molecule toxins’ limited epitopes, resolving hydrophobic aggregation, and enabling ultra-sensitive binding to trace targets (pg/mL-fM) in real matrices, thus markedly boosting screening efficiency [39,40,41]. 

## 3. SELEX Technology for Toxin-Specific Aptamer Screening

Given the structural diversity of toxin molecules—ranging from small-molecule mycotoxins (200–900 Da) to macromolecular protein toxins (30–150 kDa)—researchers have developed various specialized SELEX variants tailored to different target characteristics. SELEX technologies can be systematically classified based on the separation principle and screening strategy into five main categories: target-immobilized SELEX, library-immobilized SELEX, non-immobilized SELEX, cell-based SELEX, and High-Throughput and Automated SELEX Platforms [42,43]. 

### 3.1. Target-Immobilized SELEX

#### 3.1.1. Magnetic Beads-SELEX (MB-SELEX)

The initial utilization of magnetic beads in aptamer selection dates to 1996, when Hamm employed them to isolate aptamers binding to anti-ferritin antibody H107 [44]. In 1997, Bruno further advanced this approach by using tosyl-activated and uncoated magnetic beads (4.5 μm diameter), as well as long-chain alkylamine magnetic porous glass, to screen DNA ligands against small-molecule targets [45]. Compared to conventional SELEX methods that rely on membrane filtration, column chromatography, or microtiter plate washing—techniques characterized by low partitioning efficiency and typically requiring 10–20 selection cycles—the introduction of paramagnetic beads as solid supports enables rapid magnetic separation, eliminating labor-intensive centrifugation or filtration steps [46,47]. This approach offers several key advantages: low sample consumption (50–250 μL), stringent washing with minimal loss of bound sequences, and direct PCR amplification from bead surfaces without additional elution steps. Moreover, magnetic beads are commercially available in a wide size range (0.04–70 μm) with diverse surface functional groups (amine, carboxylic acid, tosyl, epoxy, streptavidin, Ni-NTA), providing flexible conjugation strategies tailored to specific target molecules [48].

For toxin detection, MB-SELEX has been extensively applied to both small-molecule toxins and large protein toxins. Small-molecule examples include aflatoxin B2 (AFB2) using amine-functionalized beads (10 rounds) [49], saxitoxin (STX) conjugated to a carrier protein and immobilized via epoxy groups [50]. For large protein toxins, MB-SELEX has yielded aptamers against staphylococcal enterotoxin (14 rounds) [51]. Selected aptamers typically exhibit dissociation constants in the nanomolar range. However, MB-SELEX has inherent limitations. Random target immobilization may obscure critical epitopes, generating aptamers that recognize the immobilized target rather than its native conformation. High target density on bead surfaces can induce multivalent effects, leading to overestimated affinity (apparent Kd lower than true values) and potentially selecting aptamers that fail to bind the target in homogeneous solution [52]. To address these limitations, advanced MB-related technologies have been developed, including Capture-SELEX [53], FluMag-SELEX [54], and microfluidic SELEX [55].

#### 3.1.2. FluMag-SELEX (Fluorescence Magnetic Bead-SELEX)

A seminal advancement in SELEX technology was reported by Bruno in 1997, who introduced the combined use of magnetic microbeads for target immobilization and fluorescence detection to monitor nucleic acid binding against the chloroaromatics 4-chloroaniline, 2,4,6-trichloroaniline, and pentachlorophenol [45]. FluMag-SELEX was formally introduced by Stoltenburg et al. in 2005 as an advancement of MB-SELEX that incorporates fluorescent labeling for quantitative monitoring of the selection process [54]. This technique combines target immobilization on magnetic beads with fluorescent labeling of the oligonucleotide library via labeled primers during PCR amplification, enabling quantitative monitoring of aptamer enrichment throughout the selection process. Key advantages include rapid magnetic separation, precise tracking of selection progression, and the ability to calculate enrichment factors for optimizing screening stringency, while avoiding the use of radioactive labeling. 

FluMag-SELEX has been successfully applied to the selection of aptamers against several toxin-related targets, including polychlorinated biphenyls (PCBs) [56], a class of environmental toxins, with dissociation constants in the micromolar range and detection limits as low as 0.1 ng/mL. Additionally, the Helmholtz Centre for Environmental Research (UFZ) has reported the application of FluMag-SELEX for the selection of aptamers against toxins, bacterial lipopolysaccharides (LPS), and protein A from Staphylococcus aureus [54]. However, compared to other SELEX formats such as GO-SELEX and Capture-SELEX, the application of FluMag-SELEX for small-molecule toxin aptamer selection remains relatively limited, with the majority of reported studies focusing on protein or macromolecular targets. This is partly because target immobilization—a prerequisite for FluMag-SELEX—can alter the surface chemistry of small-molecule toxins, potentially compromising the selection of aptamers that recognize the native analyte [57]. Nonetheless, FluMag-SELEX remains a valuable platform for aptamer selection against protein toxins and macromolecular targets where immobilization does not interfere with native conformation.

### 3.2. Library-Immobilized SELEX

In library-immobilized SELEX, the oligonucleotide library is anchored to a solid support, and target binding induces conformational changes that release bound sequences.

#### 3.2.1. Capture SELEX

Capture-SELEX, as the library-immobilized SELEX, is partially derived from FluMag-SELEX [54], which is characterized by target immobilization on magnetic beads and fluorescent labeling of oligonucleotides for quantification [58]. The key distinction lies in the presentation of the target and the oligonucleotides: Capture-SELEX was developed to select aptamers against targets unsuitable for immobilization on solid surfaces, such as small organic molecules, by enabling immobilization of the oligonucleotide library rather than the target. This design was adapted from the structure-switching aptamer concept [59], in which the library is anchored to a solid support via a complementary docking sequence, and target binding induces conformational changes that release bound sequences. In Capture-SELEX, the library is immobilized on streptavidin-coated magnetic beads via a biotinylated capture probe, and target-induced conformational switching releases high-affinity aptamers into solution, while unbound sequences are removed by magnetic separation [53]. This library-immobilized strategy preserves the native conformation of small-molecule targets and typically yields short-chain aptamers (approximately 40 nucleotides) with well-defined secondary structures, facilitating post-SELEX truncation and optimization. Notably, increasing the length of the capture probe can enhance selection stringency and promote the enrichment of higher-affinity aptamers [60].

Capture-SELEX has proven particularly effective for generating aptamers against small-molecule toxins that are challenging to immobilize due to limited functional groups or hydrophobicity. A high-affinity aptamer against tetrodotoxin (TTX) was selected after 23 rounds, with Kd = 25 nM as determined by bead-based colorimetric assay [61]. Aptamers against the mycotoxin zearalenone (ZEN) were obtained after 8 rounds of Capture-SELEX, exhibiting a Kd of 15.2 ± 3.4 nM [62]. Additionally, a DNA aptamer against the marine biotoxin gymnodimine-A (GYM-A) was selected, and after truncation, the aptamer G48nop achieved a Kd of 95.30 nM [63]. Recent studies have extended the application of Capture-SELEX to a broader range of toxins. In 2025, Ebanks et al. reported the selection of a DNA aptamer against AFB1 using Capture-SELEX, achieving a Kd of 42.1 ± 23.8 nM, and subsequently developed a lateral flow assay for detection in peanut extract [64]. Sun et al. successfully isolated high-affinity aptamers against azaspiracid-2 (AZA-2) with Kd values as low as 68 nM after optimization [65]. Additionally, Lock demonstrated the rapid selection of aptamers against T-2 and HT-2 mycotoxins using an improved Capture-SELEX protocol coupled with high-throughput sequencing, significantly reducing selection time [66]. 

Despite its advantages, Capture-SELEX suffers from inherent limitations, including low release efficiency of bound sequences, non-specific binding to the capture matrix, false positives due to dynamic dissociation equilibrium, and reduced library diversity [58]. To mitigate these drawbacks, strategies such as increasing capture probe length to enhance selection stringency, incorporating counter-selection steps, and coupling with high-throughput sequencing for real-time monitoring of enrichment dynamics have been successfully employed. Furthermore, integration with microfluidic platforms has enabled more precise control over selection conditions, reducing non-specific retention and improving overall efficiency. 

#### 3.2.2. GO-SELEX (Graphene Oxide-SELEX)

GO-SELEX was first introduced by Park et al. in 2012 for aptamer selection against nicotinamide phosphoribosyl transferase (NAMPT) [67]. The technique employs graphene oxide (GO) as an adsorption medium. GO is a two-dimensional carbon nanomaterial rich in oxygen-containing functional groups (epoxy, carboxyl, hydroxyl) that adsorbs free ssDNA through π-π stacking interactions between nucleic acid bases and the hexagonal carbon units, while double-stranded DNA remains unadsorbed due to its buried bases. During selection, GO adsorbs unbound ssDNA but not target-bound sequences that undergo conformational changes, allowing facile separation by centrifugation. To increase selection pressure, three operational modes have been developed: the basic mode (target incubated with library before GO addition), the adversarial mode (GO added first to immobilize the library, then target competes for binding), and the competitive mode (incorporating target analogs to eliminate cross-reactive sequences). In this library-immobilized format, GO serves as a solid support that adsorbs the ssDNA library, and target binding releases aptamers from the GO surface [68]. This approach preserves the native conformation of targets, offers simplicity and rapid operation, and circumvents the need for target modification.

GO-SELEX has been successfully applied to the selection of DNA aptamers for a wide range of small-molecule toxins, including mycotoxins, marine toxins, and soybean toxins. For mycotoxins, high-affinity aptamers have been obtained against T-2 toxin (Kd = 20.8 ± 3.1 nM) [69] and AFM1 (Kd = 109.10 ± 6.02 nM) [70]. For marine toxins, aptamers have been selected against okadaic acid (OA) (Kd = 40 nM) [71], STX (Kd = 50.75 ± 14.97 nM) [72], and gonyautoxin 1/4 (GTX1/4) (Kd = 17.7 nM) [73]. In addition to conventional GO-SELEX targeting a single analyte, multiple SELEX strategies have been developed for simultaneous selection against multiple targets. Nguyen et al. first established multiple GO-SELEX to select aptamers against three pesticides, yielding ten distinct aptamers (Kd = 10–100 nM), including cross-reactive sequences capable of binding two or three targets simultaneously [74]. Subsequently, Gu et al. developed magnetic reduced graphene oxide (MRGO)-SELEX for multiplex selection against three marine toxins, obtaining specific aptamers for each toxin as well as multitarget aptamers capable of binding either domoic acid(DA) or TTX [75]. 

However, spontaneous desorption of weakly bound sequences can occur during the separation step of GO-SELEX, potentially leading to contamination of the enriched pool and incomplete removal of unbound ssDNA. Additionally, residual GO particles may interfere with downstream applications such as PCR amplification or sensor fabrication, necessitating careful purification steps. The adsorption efficiency can also be influenced by the physicochemical properties of the target molecules, and certain targets may not induce sufficient conformational changes for effective desorption from the GO surface [68,74]. Despite these limitations, GO-SELEX remains a powerful and versatile platform for generating high-affinity aptamers against small-molecule toxins and other targets where immobilization is challenging.

#### 3.2.3. GNP-SELEX (Gold Nanoparticle-Assisted SELEX)

Similar to GO-SELEX, GNP-SELEX operates as a library-immobilized platform. It relies on the preferential adsorption of the ssDNA library onto gold nanoparticles (GNPs) via electrostatic and van der Waals interactions. In a typical GNP-SELEX workflow, the ssDNA library is first immobilized on the GNP surface. In the absence of a target, the adsorbed ssDNA stabilizes GNPs against salt-induced aggregation. Upon addition of the target, specific binding between the target and the immobilized ssDNA induces a conformational change, which triggers the desorption (release) of the aptamer from the GNP surface. The liberated GNPs are then susceptible to aggregation in the presence of salt (e.g., NaCl), producing a visible color change from red to purple. This target-induced release mechanism enables real-time visual monitoring of aptamer enrichment without complex instrumentation, while keeping the target immobilization-free and thus preserving its native conformation. 

The development of GNP-based SELEX was motivated by the need to address residual binding—a phenomenon in which aptamers remain adsorbed on GNPs even after target binding, compromising sensitivity [76]. Independently, Chatterjee et al. developed GOLD SELEX, in which the library is first incubated with GNPs, and only sequences with binding affinity greater than their affinity for GNPs are released upon target addition, thereby improving aptamer sensitivity [77]. Building upon this principle, Lee et al. subsequently established GNP-SELEX as a visual, self-monitoring platform for small-molecule aptamer discovery. Through alternating positive and negative selection rounds monitored by the extinction ratio (E520/E700), this method enables rapid determination of target-specific aptamer enrichment without target modification or post-SELEX analysis such as qPCR [78].

As a recently developed platform, GNP-SELEX has thus far been applied to a limited but growing range of small-molecule targets. For brassinolide (BL) and bisphenol A (BPA), Lee et al. identified aptamers gBL9-20 (Kd = 32.9 nM) and gBPA11-40 (Kd = 58.7 nM) after 9 and 11 rounds, respectively. Through 3D molecular simulation and truncation, they obtained enhanced aptamers tgBL-v2 (Kd = 17.3 nM) and tgBPA-v2 (Kd = 37.9 nM), which were used to develop an aptamer-mediated precipitation assay for BL detection in plant extracts (limit of detection(LOD) = 0.6 pg/mL) and a colorimetric DNAzyme-based assay for BPA detection on thermal receipt paper (LOD = 0.3 ng/mL), respectively [78].

Despite its advantages, GNP-SELEX has inherent limitations. Ding et al. reported that during aptamer selection against adenosine, the amount of DNA released from GNPs was extremely low, with most ssDNA remaining adsorbed [79]. This strong adsorption may prevent certain small-molecule targets from binding to ssDNA or may retain bound aptamers on the GNP surface, limiting the applicability of GNP-SELEX for targets with weak binding affinity [76,80]. Additionally, the technique may be unsuitable for targets that possess intrinsic affinity for GNPs themselves, as this can interfere with the selection process. Excessive non-specific adsorption of non-target sequences and low DNA release efficiency (<1%) in the presence of target have also been observed, and the technique may be unsuitable for certain protein targets due to direct protein-GNP interactions [48].

### 3.3. Non-Immobilized SELEX

CE-SELEX (Capillary Electrophoresis-SELEX) was pioneered by Mendonsa and Bowser in 2004, who successfully screened aptamers against human IgE in just four rounds [81]. The technique utilizes capillary electrophoresis to separate bound and unbound sequences based on differences in electrophoretic mobility in free solution. Target molecules and the oligonucleotide library interact in their native conformations, and target-aptamer complexes migrate faster than free ssDNA, enabling efficient separation within minutes per round. This approach offers high resolution, rapid separation, and exceptional screening efficiency, often reducing selection rounds to 1–4 compared to 10–20 rounds in conventional methods [82]. Several key advantages distinguish CE-SELEX from immobilized SELEX methods. It operates entirely in free solution, eliminating the need for target or library immobilization and avoiding structural masking. When coupled with fluorescence-labeled libraries, it enables real-time visual monitoring of complex formation and enrichment dynamics. The technique also eliminates the need for negative selection steps [83], and its nanoliter injection volume minimizes consumption of the library and target, making it particularly suitable for precious or limited samples.

CE-SELEX has been extensively applied to protein targets, including neuropeptide Y (NPY), histone H4-K16Ac, glycosylated VEGF peptide fragments, CRISPR/Cas9, etc. demonstrating its versatility for diverse biomolecular targets [84,85,86,87]. Although the application of CE-SELEX for toxin detection remains limited, Tang et al. successfully applied CE-SELEX to screen aptamers against ricin toxin. After only four rounds of selection, the enriched pool exhibited 87.2% binding to ricin, and high-affinity aptamers were obtained with Kd of 58 nM and 80 nM [88].

Despite its advantages, the application of CE-SELEX for small-molecule toxin aptamer selection remains limited due to technical challenges. Direct visualization of target-aptamer complexes is difficult, making accurate collection challenging. Separation efficiency is highly sensitive to solution properties (ionic strength, pH), which may conflict with optimal binding conditions. The nanoliter injection volume limits library diversity, potentially leading to incomplete recovery of bound sequences. Additionally, the small electrophoretic mobility difference between complexes and free ssDNA can hinder effective separation. CE-SELEX also requires specialized instrumentation and technical expertise [89]. To address these challenges, a low-pH CE-SELEX (LpH-CE-SELEX) variant was developed. By lowering the separation buffer pH to 2.6, proteins become positively charged while ssDNA remains negatively charged, enabling oppositely directed migration and facilitating convenient collection of protein-ssDNA complexes [90].

### 3.4. Cell SELEX/WC-SELEX (Whole Cell SELEX)

In contrast to conventional SELEX methods that require purified target molecules, Cell-SELEX (also known as whole cell-SELEX) utilizes whole living cells as screening targets, preserving membrane proteins and surface markers in their native state with natural post-translational modifications. The application of SELEX to cellular targets was first demonstrated by Morris et al. (1998) using red blood cell membranes [91]. Subsequently, Daniels et al. (2003) extended this approach to whole living cancer cells, establishing the methodology now widely known as whole cell-SELEX [92]. The technique was further refined and popularized by Shangguan et al. (2006) under the leadership of Weihong Tan, who successfully selected aptamers against various cancer cells using whole cell-SELEX [93,94]. A typical Cell-SELEX workflow involves alternating rounds of negative and positive selection. In the negative selection step, the ssDNA library is incubated with counter-target cells to remove non-specific binders; the unbound sequences are then incubated with target cells during the positive selection step, allowing specific aptamers to bind to their cognate surface epitopes. After washing, bound aptamers are eluted by heat denaturation, amplified by PCR, and converted to ssDNA for subsequent rounds. Typically, 10–20 rounds of selection are required to achieve high affinity and specificity [95]. This approach offers several key advantages over target-purified SELEX: it maintains the native conformation of cell surface targets, enables simultaneous discovery of aptamers against multiple unknown surface markers, and circumvents the need for target purification—particularly valuable for membrane proteins and other difficult-to-express targets.

Cell-SELEX was originally driven by the need for molecular probes in cancer research, enabling the selection of aptamers that recognize tumor cell surface markers without prior knowledge of their molecular identities—a capability that has since been extended to the detection of toxin-producing bacteria and a parasitic pathogen [96]. For Bacterial pathogens, an early example of applying SELEX to whole bacterial cells was demonstrated by Bruno and Kiel, who successfully selected DNA aptamers against Bacillus anthracis spores using autoclaved spores as targets in 1999 [97]. Song et al. (2019) employed Cell-SELEX combined with functionalized graphene oxide and rolling circle amplification to obtain highly specific aptamers against Vibrio parahaemolyticus (Kd = 10.30 ± 2.50 nM) [98]. For a parasitic pathogen, Nagarkatti et al. (2012) developed RNA aptamers using whole-cell SELEX against live trypomastigotes of Trypanosoma cruzi (Kd = 7.68 ±1.63 nM) [99].

Despite its utility for pathogen detection, Cell-SELEX is less suitable for direct selection against free toxins, as most toxins are secreted soluble molecules not anchored to the cell surface. Additional limitations include high library complexity, cell-to-cell variability, the risk of selecting aptamers against non-toxin surface proteins, and the labor-intensive requirement of maintaining viable cells [96]. To address these challenges, improved platforms such as FACS-assisted, 3D culture-based, nanomaterial-assisted, and microfluidic chip-based Cell-SELEX have been developed [100,101,102], and the evolution toward tissue-SELEX offers a more physiologically relevant approach [103]. However, for direct free toxin detection, non-immobilized SELEX and library-immobilized methods are generally more suitable.

### 3.5. High-Throughput and Automated SELEX Platforms

Unlike the above categories defined by target-library interaction modes, the methods described in this section are technological enhancements that can be combined with immobilization-based or immobilization-free SELEX to improve efficiency and throughput.

#### 3.5.1. Microfluidic SELEX

Microfluidic SELEX integrates the entire aptamer selection process into microfabricated chips, enabling automated, high-throughput screening with minimal reagent consumption and precise control over fluid dynamics, incubation time, and separation efficiency [104,105,106]. The concept was pioneered by Hybarger et al. in 2006, who developed the first microfluidic-based automated SELEX prototype [107]. Since then, microfluidic platforms have revolutionized aptamer selection by automating labor-intensive steps such as mixing, incubation, washing, and amplification, significantly reducing human intervention and experimental variability [108]. Several key advantages distinguish microfluidic SELEX from conventional methods. First, the miniaturized system dramatically reduces sample and reagent consumption (from milliliters to microliters), lowering costs and enabling screening of precious targets. Second, precise control over flow rates, incubation times, and shear forces during washing steps enhances selection stringency and reproducibility. Third, the integration of multiple functions—including mixing, incubation, separation, and amplification—onto a single chip enables automation and parallel processing, eliminating manual errors and cross-contamination risks. Fourth, the ability to incorporate negative selection and counter-selection steps on-chip improves aptamer specificity. As a result, microfluidic SELEX typically requires only 5–8 rounds (compared to 10–20 rounds for conventional SELEX) and can be completed within hours or days rather than months [105,109,110,111].

Microfluidic SELEX has been successfully applied to screen aptamers against various classes of toxins, including cyanotoxins, mycotoxins, marine toxins, environmental pollutants, and pesticide residues. For the cyanotoxin microcystin-LR (MC-LR), a potent hepatotoxin harmful to human health, a microfluidic aptasensor system using a fluorescence-tagged aptamer achieved a LOD = 1.9 ppb, significantly lower than the World Health Organization safety threshold for recreational waters [112]. For the mycotoxin ochratoxin A (OTA), a lab-on-chip aptasensor achieved a LOD of 1.3 ng/mL over a detection range of 5–200 ng/mL [113], while a graphene field-effect transistor-based microfluidic aptasensor reached an ultra-low LOD of 4 pg/mL (10 pg/mL to 4 ng/mL) [114]. For AFM1, a class I carcinogen found in milk, a microfluidic paper-based analytical device (μPAD) achieved an LOD of 3 pM in buffer and 10 nM in milk [115]. For OA, an electrochemical microfluidic biochip achieved an LOD of 8 pM with a linear detection range of 10–250 nM [116]. For PCBs, an environmental toxin, a microfluidic electrochemical aptasensor achieved an ultra-low LOD of 0.16 fg/mL over a broad detection range of 0.1 pg/mL to 1000 ng/mL [117]. For TTX, microfluidic-based platforms have achieved detection limits in the nanomolar range [104]. For pesticide residues, a smartphone-integrated microfluidic aptasensor using gold-coated polystyrene microparticles achieved rapid, label-free detection of imidacloprid and carbendazim with limits of detection of 3.12 ppm and 1.56 ppm, respectively [118].

Nevertheless, despite these advances, several limitations of microfluidic SELEX remain, including complex chip fabrication requiring specialized expertise, bead aggregation within microchannels, and challenges in scaling up for clinical applications. To address these challenges, several emerging directions promise to further enhance the platform. Conditional SELEX enables aptamer selection under varying ion concentrations, pH, and temperature to maintain functionality in physiological environments [110]. In vivo-like SELEX simulates complex biological conditions such as the tumor microenvironment or patient blood, selecting aptamers that retain high affinity in biological fluids [119]. Additionally, non-SELEX methods on microfluidic chips eliminate PCR amplification bias by relying solely on high-resolution partitioning techniques [120]. With continued advances in chip design, automation, and bioinformatics tools such as AlphaFold, microfluidic SELEX is expected to play an increasingly important role in rapid aptamer discovery for toxin detection and other applications.

#### 3.5.2. High-Throughput SELEX (HT-SELEX)

HT-SELEX was first introduced by Zhao et al. in 2009, who coined the term and demonstrated that a single round of selection combined with high-throughput sequencing could accurately infer binding energies, outperforming standard motif-finding algorithms [121]. This established HT-SELEX as a quantitative platform for dissecting the energetic basis of molecular recognition, rather than merely a screening tool for high-affinity sequences [121]. The method was subsequently popularized by Jolma et al. (2010), who applied massively parallel sequencing to simultaneously characterize the DNA-binding specificities of multiple transcription factors [122]. Compared to conventional SELEX, HT-SELEX offers several key advantages. Traditional SELEX requires 9–20 rounds over 2–3 months, while HT-SELEX enables deep sequencing after each round to track sequence enrichment dynamics, allowing identification of high-affinity candidates with significantly reduced selection rounds (typically <8 rounds) and total time within 3 weeks. By generating comprehensive sequence enrichment profiles across selection rounds, HT-SELEX enables the mapping of binding affinity landscapes across sequence space, the identification of conserved motifs critical for target recognition, and the systematic assessment of sequence variations on binding function.

For toxin detection, the application of HT-SELEX has grown in recent years. Shareef and Hariprasad (2025) combined HT-SELEX with AuNP-SELEX to screen aptamers against AFB1, identifying aptamer L2 with a Kd of 1.24 nM and a fluorescence assay achieving an LOD of 0.53 nM [123]. For bacterial toxin detection, Oliveira et al. (2024) applied HT-SELEX to staphylococcal enterotoxin A (SEA), analyzing approximately 3 million aptamer candidates after 10 rounds of selection and identifying an aptamer with a Kd = 13.36 ± 18.62 nM [124].

Despite its advantages, HT-SELEX requires high-throughput sequencing infrastructure, substantial computational resources, and specialized expertise in NGS data processing. Additionally, the high cost of NGS may limit its accessibility for routine laboratory use, and robust bioinformatics pipelines are essential to distinguish true enrichment from PCR bias and amplification artifacts. As demonstrated by Takahashi et al. (2016), careful optimization of amplification conditions remains critical, as different PCR methods (e.g., conventional solution PCR versus droplet digital PCR) can lead to divergent selection outcomes [125]. Furthermore, extensions such as SNP-SELEX have highlighted the complexity of interpreting sequence variants, underscoring the need for advanced computational models to accurately assess binding effects [126]. Beyond aptamer discovery, HT-SELEX has evolved into a platform for mechanistic exploration. Mukherjee et al. (2021) combined HT-SELEX with bioinformatic motif analysis (RaptRanker) to identify the sequence-structure motifs of pre-miRNA responsible for binding to a small-molecule ligand, providing mechanistic insights into RNA–small molecule interactions and miRNA biogenesis [127]. Most recently, the integration of artificial intelligence with HT-SELEX has enabled the de novo design of functional nucleic acids, as exemplified by the InstructNA framework, which generated significantly stronger binders compared to traditional HT-SELEX [128].

The key features, advantages, and limitations of the nine SELEX variants discussed above are summarized in Table 1.

Different modified SELEX variants are not simply superior or alternative to one another; instead, they constitute a combinatorial toolkit selected according to target molecular weight, screening objectives and experimental resources [129]. For macromolecular protein toxins, MB-SELEX and CE-SELEX deliver outstanding performance owing to their efficient separation capacity and favorable preservation of the native target conformation [130]. For small-molecule toxins, library immobilization strategies including Capture-SELEX, GO-SELEX and GNP-SELEX are preferred, as they evade epitope masking induced by target immobilization [131]. In terms of screening efficiency, microfluidic SELEX and CE-SELEX can shorten the traditional multi-month selection procedure to days or even hours, yet they are limited by complicated chip fabrication and restricted library coverage from nanoliter-scale injection volumes, respectively [96]. Although HT-SELEX enables affinity profiling across the full sequence space via deep sequencing, it suffers from PCR amplification bias and substantial sequencing costs [125]. In recent years, researchers have moved beyond optimization of individual SELEX platforms toward integrated cross-strategies. Representative combinations include coupling high-throughput sequence outputs from HT-SELEX with rapid visual validation via GNP-SELEX, or implementing preliminary enrichment on microfluidic chips followed directly by molecular docking simulation [123,132].

## 4. Challenges and Perspectives

### 4.1. Key Challenges in Toxin Detection

Despite the significant advances in SELEX technology over the past three decades as Table 2 displayed, several key challenges remain in its application to toxin detection, particularly for ECs of trace-level presence and complex environmental fate. These challenges span fundamental limitations in small molecule recognition, practical hurdles in complex sample matrices, technical difficulties specific to RNA aptamers, and the trade-offs associated with high-throughput screening platform.

- Challenges in small-molecule toxin aptamer selection. Although SELEX has been successfully applied to a wide range of small-molecule toxins, the intrinsic properties of small molecules—limited epitopes and fewer interaction sites—often make it more challenging to achieve ultra-high affinity compared to protein targets [57]. Their interactions with nucleic acid aptamers primarily rely on weak forces such as hydrogen bonding, hydrophobic interactions, and π-π stacking, rather than the extensive contact interfaces typical of protein-aptamer complexes. Immobilization-free SELEX methods have largely overcome the conformational issues associated with target immobilization, yet achieving picomolar-level affinity for small molecules remains demanding.
- Anti-interference ability in complex matrices. Real samples (e.g., food extracts, environmental water, serum) contain abundant interfering substances (lipids, proteins, polysaccharides) that may non-specifically bind to non-target sequences or compete with the target for aptamer binding sites. Although negative selection and counter-selection can improve specificity, maintaining high affinity and selectivity in complex matrices remains a major challenge, with matrix effects often reducing detection sensitivity by more than an order of magnitude in complex samples such as milk, beer, coffee, and wine [133,134].
- Technical challenges specific to RNA aptamer selection. RNA aptamers offer unique structural diversity and can be engineered into functional nucleic acids such as ribozymes and aptazymes, enabling label-free signal amplification and providing valuable tools for studying RNA-small molecule interactions. However, their application in toxin detection remains limited due to technical challenges: RNA is highly susceptible to nuclease degradation, the selection process involves reverse transcription and in vitro transcription, making it more time-consuming and costly than DNA SELEX, and library synthesis biases can affect selection outcomes [125]. Nevertheless, recent studies have demonstrated the potential of RNA aptamers, including the identification of pre-miRNA binding motifs for small-molecule ligands and the development of fluorogenic RNA-based biosensors using droplet microfluidics [127].
- Selection efficiency and cost. Traditional SELEX requires 10–20 rounds over 2–3 months, consuming substantial amounts of reagents and labor. Conventional SELEX fails to identify the optimal aptamer sequence [38,47,135]: (1) Initial libraries have insufficient molecular diversity to cover all potential high-affinity sequences; (2) Complex folded high-affinity oligonucleotides suffer poor PCR amplification and get lost during repeated selection cycles; (3) SELEX only enriches locally functional sequences rather than globally optimal ones, confirmed by post-SELEX truncation/mutation tests. As the demand for rapid, high-throughput, and cost-effective aptamer discovery grows, particularly for toxin detection in complex environmental and food samples, the limitations of conventional SELEX have become increasingly apparent. To address these challenges, HT-SELEX and microfluidic SELEX have emerged as powerful alternatives, significantly improving efficiency by reducing selection rounds to typically <8 and time to days or hours [104,105,106]. However, these advanced platforms introduce new challenges of their own. The instrumentation required for high-throughput sequencing remains expensive, and the associated computational resources and bio-informatics expertise add to the overall cost, limiting accessibility for routine laboratories. Furthermore, processing millions of sequences to distinguish true enrichment from PCR bias requires sophisticated algorithms and careful experimental design. Different PCR methods (e.g., conventional solution PCR vs. droplet digital PCR) can lead to divergent selection outcomes, further complicating data interpretation and underscoring the need for standardized protocols [125].

### 4.2. Future Directions

To overcome the inherent limitations of individual SELEX platforms, researchers have increasingly developed integrated and multidimensional strategies. These include novel nanomaterials (GO, GNPs, magnetic beads), multi-target selection, and microfluidic automation. Beyond these material and methodological innovations, the convergence of AI with HT-SELEX is now reshaping aptamer discovery from a screening-driven process into a design-driven discipline. The following sections discuss these emerging paradigms.

#### 4.2.1. Material and Methodological Innovations

- Material innovations. The introduction of novel nanomaterials has revolutionized SELEX by providing new separation mechanisms and signal transduction modes. GO serves as an efficient adsorption medium for immobilization-free selection, enabling aptamer screening against T-2 toxin [69], OA [71], and GTX [73]. GNPs offer colorimetric readout, allowing real-time visual monitoring of library enrichment via extinction ratio changes without complex instrumentation; this platform has been successfully applied to BL and BPA [78]. Magnetic nanoparticles facilitate rapid separation and have been integrated into multiple SELEX formats, including MB-SELEX and MRGO-SELEX, for efficient aptamer selection against marine toxins [75].
- Methodological innovations. A range of strategies have been developed to expand the scope and efficiency of SELEX. Multi-target selection challenges the traditional “one-target-one-SELEX” paradigm by enabling simultaneous isolation of aptamers against several targets in a single campaign. Using multiplex GO-SELEX, Nguyen et al. obtained ten distinct aptamers (Kd = 10–100 nM) against three pesticides, including cross-reactive sequences capable of binding two or three targets [74]. Building on these methodological advances, multi-target selection strategies have enabled a distinct application: the development of broad-spectrum aptamers capable of recognizing entire families of structurally related toxins. Such strategies are particularly valuable for toxin families with structural similarity, offering a pathway toward broad-spectrum recognition elements.

Beyond multi-target selection, the integration of complementary technologies has emerged as a powerful paradigm and has been successfully applied to various toxins. For example, the combination of HT-SELEX with AuNP-SELEX, which leverages high-throughput sequencing and nanomaterial-based selection to streamline aptamer discovery [123]; the integration of microfluidics with magnetic beads, which automates selection, reduces rounds, and introduces tunable shear forces for precise stringency control [104,105,106]; the coupling of Capture-SELEX with droplet microfluidics for RNA aptamer selection with functional readout [136]; and the high efficiency of CE-SELEX, which achieves significant enrichment within minimal rounds [88], etc.

Chaoyong James Yang’s team have contributed two notable innovations that bridge SELEX methodology and downstream sensor integration. First, they developed monoclonal surface display SELEX (MSD-SELEX) based on single-molecule emulsion PCR and agarose bead separation, enabling simple, rapid, and cost-effective aptamer enrichment without large-scale DNA sequencing; this method was successfully applied to AFB1 [137]. Second, they engineered an AFB1-responsive hydrogel integrated with a distance-readout microfluidic chip, allowing portable visual quantitative detection of AFB1 in real samples [138]. These examples illustrate how methodological advances in SELEX can be directly translated into user-friendly detection devices.

Collectively, these trends reflect a transition from method-oriented to demand-oriented aptamer development, where selection workflows are tailored to target characteristics and downstream application requirements-a paradigm further advanced by the convergence of artificial intelligence with HT-SELEX for de novo aptamer design.

#### 4.2.2. AI-Assisted and Computational Design

**Figure 3:**
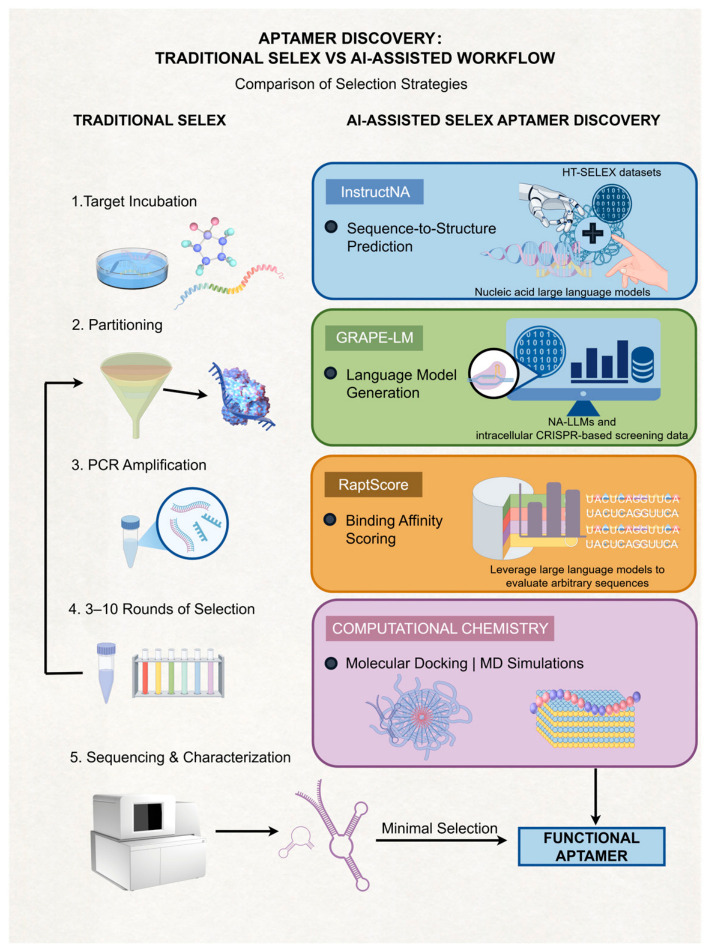
AI-driven aptamer discovery paradigm versus traditional SELEX workflow. Schematic comparison of aptamer discovery strategies. **Left**: Traditional SELEX workflow, **Right**: AI-assisted aptamer discovery, featuring InstructNA for sequence-to-structure prediction, GRAPE-LM for de novo aptamer sequence generation, and RaptScore for binding affinity evaluation. Complemented by computational chemistry approaches including molecular docking and molecular dynamics (MD) simulations, this design-driven strategy enables functional aptamer generation with minimal experimental selection rounds, overcoming the limitations of traditional screening methods and accelerating aptamer development. (Some drawing elements are from www.figdraw.com).

The integration of AI is reshaping aptamer discovery from a screening-driven process to a design-driven discipline, as shown in Figure 3. Traditional SELEX, even in high-throughput formats, remains constrained by the vastness of sequence space and its reliance on experimental enrichment. AI-assisted approaches address these limitations by learning sequence-function relationships from HT-SELEX data and enabling de novo design without prerequisite structural information.

A representative advance is the InstructNA framework, which combines nucleic acid large language models (NA-LLMs) with HT-SELEX datasets to generate functional aptamers directly from sequence information, producing significantly stronger binders than conventional HT-SELEX [128]. Complementary AI strategies have further expanded this paradigm. GRAPE-LM integrates NA-LLMs with intracellular CRISPR-based screening data to achieve single-round evolution of RNA aptamers [139]. SILEX is a novel single-round aptamer screening strategy reported in 2026. Different from multi-cycle SELEX, it only executes one incubation round, utilizes UMI tags to eliminate PCR bias, and adopts UAE-Clustering machine learning to mine conserved binding core structures from sequencing data. Initial single-round libraries retain complete motif information equivalent to multi-round enriched pools. Guided by predicted secondary structures, aptamers can be truncated and mutated to achieve affinity improvement, greatly shortening screening cycles and avoiding loss of optimal sequences caused by repeated amplification [140]. RaptScore leverages large language models to evaluate arbitrary sequences—including those absent from SELEX—accommodating variable sequence lengths and demonstrating strong correlation with binding activity; when integrated with in silico maturation, it enables truncation by up to 10 nucleotides while maintaining binding efficiency [141].

Complementary to AI-assisted analysis of HT-SELEX data, Huang and co-workers have pioneered computational strategies that bypass or complement experimental SELEX for marine toxin aptamer discovery. One innovation is a “docking-then-assembling” approach, which docks individual nucleotides to OA and assembles them into full aptamer sequences, enabling de novo design without prior SELEX enrichment [142]. Another strategy repurposes existing thermally stable DNA aptamers by using molecular docking to screen for toxin targets. Unlike classic virtual screening that computationally scans random sequence pools of enormous size, this repurposing approach restricts the candidate space to a library of structurally validated, thermally stable DNA aptamers, followed by docking-based screening against TTX and simulation-guided engineering to enhance affinity and specificity [143]. Collectively, these studies exemplify how computational chemistry and molecular simulation can reduce reliance on labor-intensive screening and accelerate aptamer development for small-molecule toxins.

A typical comparative case of thrombin aptamers reported by Di Gioacchino et al. clearly differentiates sequences acquired from conventional SELEX and machine learning approaches: the traditional SELEX approach in the prior study identified the strong binders ThA (5′-AGGAGATGATGTGTGGTAGGC-3′, exosite II) and ThD (5′-GTAGGATGGGTAGGGTGGTC-3′, exosite I). In contrast, the RBM-based (AI-driven) design generated de novo sequences such as r8 (5′-GAGGGTTGGTTGGTTGGTGC-3′) and r9 (5′-AGGGTTGGTTGGTTGGTTGGC-3′), which were experimentally validated as exosite-I binders, demonstrating the model’s ability to produce functional aptamers not present in the original training data [144]. This contrast reveals inherent limitations of iterative wet SELEX: repeated PCR enrichment filters out low-abundance high-affinity clones and fails to excavate latent binding motifs, whereas generative machine learning models break the constraint of initial library diversity to predict superior binders that do not exist in the starting pool, providing a reliable strategy to obtain globally optimal aptamers beyond locally enriched sequences.

As experimental HT-SELEX datasets continue to grow, the synergy between high-throughput experimentation and AI-powered analysis is expected to become a cornerstone of next-generation aptamer discovery.

Apart from large AI models and molecular docking tools, in silico SELEX has emerged as a low-cost pre-screening strategy for rapidly mining high-specificity aptamers against toxins, drastically reducing the number of wet-lab selection rounds and reagent consumption. Conventional wet-lab SELEX relies on repeated cycles of incubation, separation and amplification to enrich candidate sequences, whereas in silico SELEX accomplishes library construction, target binding simulation, affinity scoring and in silico counter-selection to remove cross-reactive candidates entirely via computational simulation. This workflow accelerates aptamer discovery while simultaneously improving target specificity and binding affinity [145,146]. Furthermore, computational modeling enables detailed three-dimensional visualization of aptamer-target intermolecular interactions, a capability inaccessible to traditional SELEX experiments [147]. Escamilla-Gutiérrez et al. constructed a two-stage in silico screening pipeline integrating sequence-based RPISeq prediction and multi-program consensus molecular docking. By performing structural clustering on large virtual nucleic acid libraries, they successfully identified specific RNA aptamers targeting three critical bacterial warfare toxins: staphylococcal enterotoxin B (3SEB), cholera toxin (1XTC) and botulinum toxin (3BTA) [148]. Rasouli Jazi et al. developed a complete, original in silico SELEX framework to screen high-specificity RNA aptamers against the outer membrane protein OmpU of *Vibrio cholerae*. Molecular dynamics (MD) simulations validated the stability of aptamer-protein complexes for rapid cholera diagnosis, marking the first report of an in silico aptamer screening strategy targeting *V. cholerae* OmpU [149]. Nevertheless, artificial intelligence approaches carry inherent limitations. Their predictive reliability heavily relies on the quality and scale of training datasets (typically deep sequencing outputs from HT-SELEX). For toxin targets lacking abundant labeled binding and non-binding sequence data, AI-driven prediction may suffer from markedly elevated false positive rates. A balanced, rational solution lies in iterative wet-dry integration: microfluidic or HT-SELEX experiments rapidly generate preliminary enrichment profiles, AI models extrapolate unexplored sequence spaces and predict critical functional mutations, and low-throughput wet assays conduct targeted validation, achieving an optimal trade-off between screening efficiency and predictive accuracy.

#### 4.2.3. Aptamer Engineering for Toxin Detection

Despite successful SELEX screening, practical toxin detection faces four bottlenecks: low affinity for small molecules, nuclease degradation in complex matrices, insufficient sensitivity for trace toxins, and poor signal transduction. Post-SELEX engineering—encompassing cyclization, multivalent assembly, and structure-switching design—provides systematic solutions to these challenges.

Cyclization improves both affinity and stability. Tan’s group developed photochemical covalent locking of aptamers, reducing binding energy about 1.4-fold for small-molecule toxins [150]. Li’s team first pioneered the direct selection of circular DNA aptamers from a circular DNA library, yielding two high-affinity circular aptamers against C. difficile glutamate dehydrogenase—one functional in both circular and linear forms, the other exclusively active in the circular configuration, demonstrating that circularization itself can generate unique binding functionalities [151]. Building on this, they further performed evolution directly in 100% human serum, yielding pM-affinity circular aptamers with high biostability—directly applicable to toxin detection in food or environmental matrices [152].

Multivalent assembly boosts sensitivity via avidity effects. Tan’s supramolecular strategies (e.g., gold nanocluster self-assembly) extended serum stability from 3 h to 48 h, critical for field-deployable toxin sensors [153].

Structure-switching signaling aptamers, pioneered by Li, directly couple target binding with fluorescence output, enabling antibody-free, rapid toxin detection [59]. Integration with rolling circle amplification produced a paper-based device detecting 0.1 nM C. difficile toxin B within 16 min [154].

Together, Li’s source innovations (structure-switching, circular selection) and Tan’s molecular engineering (cyclization, multivalency) directly address key bottlenecks in toxin detection.

#### 4.2.4. Other Emerging Directions

Several other directions are expanding the scope of SELEX-derived aptamers for toxin detection. RNA aptamers provide unique structural diversity, with their utility being advanced by chemical modifications and SELMA (Selection with Modified Aptamers) for bulky modifications incompatible with PCR amplification [127,155]. Meanwhile, the integration of smartphone-based detection with portable aptasensors is enabling point-of-care(POC) screening of emerging contaminants in food and environmental matrices [118].

Collectively, these directions are broadening the toolkit for generating aptamers tailored to the specific challenges of toxin detection.

## Figures and Tables

**Table 1:** Systematic comparison of nine SELEX variants across five categories for toxin detection summarized in this review.

SELEX Variant (Category)	Core Principle	Advantages	Limitations	Typical Rounds	Refs.
MB-SELEX (target-immobilized)	Target immobilization on magnetic beads, magnetic separation	Rapid separation, low sample consumption	May obscure critical epitopes	10–14	[1,2,3,4,5,6,7,8]
FluMag-SELEX (target-immobilized)	Target on magnetic beads + fluorescent labeling of the library	Rapid magnetic separation, quantitative monitoring of enrichment, avoids radioactivity	Target immobilization may alter the surface chemistry of small molecules	8–12	[9,10,11,12,13]
Capture-SELEX (library-immobilized)	Library immobilization, target-induced conformational release	Preserves the native conformation of small molecules	Low release efficiency	8–12	[14,15,16,17,18,19,20,21,22]
GO-SELEX (library-immobilized)	Library immobilized on GO, target binding induces release from the GO surface	Simple operation, no target modification, preserves target conformation	Residual GO may interfere with downstream applications	6–10	[23,24,25,26,27,28,29]
GNP-SELEX (library-immobilized)	GNPs adsorb free ssDNA, target binding induces color change	Visual monitoring, no complex instrumentation	Strong adsorption may hinder weak binders	9–11	[30,31,32]
CE-SELEX (non-immobilized)	Capillary electrophoresis separates complexes from free sequences	Few selection rounds, no immobilization	Difficult visualization, sensitive to solution conditions	1–4	[33,34,35,36,37,39,40,41]
Cell-SELEX (cell-based)	Live cells as targets, alternating positive and negative selection	Preserves the native conformation of membrane proteins	Not suitable for free toxins	10–20	[42,43,44,45,46,47,48,49,50]
Microfluidic SELEX (high-throughput platform)	Chip-integrated selection, separation, and amplification	Automated, low reagent consumption, tunable shear forces	Complex chip fabrication	5–8	[51,52,53,54,55,56,57,58,59]
HT-SELEX (high-throughput platform)	High-throughput sequencing tracks enrichment dynamics	Reduced rounds, maps binding affinity landscapes	High cost, requires bio-informatics expertise	4–8	[60,61,62,63,64,65,66]

**Table 2:** Representative aptamers selected by different SELEX methods for toxin detection.

Category	Target	SELEX Method	Sequence (5′→3′)	Affinity	Ref.
Mycotoxins	AFB1	Capture-SELEX	5′-AGCAGCACAGAGGTTCAGATGTTTTGTGGGTAGGGCGGGTTGGTTTTTCCCTATGCGTGCTACCGTGAA-3′	42.1 ± 23.8 nM	[19]
		HT-SELEX	5′-ATAGCATGAATTCCCGAAGACGCGTCCGGGCATACTTACTTATTCGGCTCCACTATTGAACCAGTCGTGCGACGAGGTCTAGAATACCAG-3′	1.24 nM	[62]
	AFB2	MB-SELEX	5′-AGCAGCACAGAGGTCAGATGACACCCTGGACCTTGGGATTCCGGAAGTTTTCCGGTACCTATGCGTGCTACCGTGAA-3′	9.83 nM	[6]
	AFM1	GO-SELEX	5′-CCTCTCTATGGGCAGTCGGTGATCCTAAGGGACTTTTGTCTCTCTGTGTCTTTTGCCGCTATGGCAGTGTGTAGGGAGAATGAGGAACCCAGTGCAG-3′	109.10 ± 6.02 nM	[27]
	T-2 toxin	GO-SELEX	5′-GTATATCAAGCATCGCGTGTTTACACATGCGAGAGGTGAA-3′	20.8 ± 3.1 nM	[23]
	ZEN	Capture-SELEX	5′-ATACCAGCTTATTCAATTGGCGAGATTATGAGGCTCGATCACGACAATGGTGGGGTATGCGAAAGATGAACCATAGTGGGACAATCGTAATCAGTTAG-3′	15.2 ± 3.4 nM	[22]
Marine toxin	OA	GO-SELEX	5′-ATTTGACCAT GTCGAGGGAG ACGCGCAGTC GCTACCACCT-3′ (truncated)	40 nM	[26]
	STX	GO-SELEX	5′-TTTTTTAGGGAAGAGAAGGACATATGATGGGCACAAGGCCCTCATCAATCGGTATACGGGTTGACTAGTACATGACCACTTGA-3′	50.75 ± 14.97 nM	[28]
	TTX	Capture-SELEX	5′-ATACCAGCTTATTCAATTTGAGAAAATATGAGGCTCGATAAAAAATAATAGTATAGAAATATATAAAGTGGTATTTTGAGATAGTAAGTGCAATCT-3′	25 nM	[17]
	GTX1/4	GO-SELEX	5′-AACCTTTGGTCGGGCAAGGTAGGTT-3′ (truncated)	17.7 nM	[29]
	GYM-A	Capture-SELEX	5′-GCGACCGAAAGTGAGGCCTCGATCCAAGGTGGACGGGAGGTGTGGATTGTGCGTG-3′ (truncated)	95.30 nM	[21]
	AZA-2	Capture-SELEX	5′-ACTAGGGCAAATCTATACAGTGCCAATTTCA-3′	68 nM	[20]
Bacterial toxin	SEA	HT-SELEX	5′-ATTTGCAATACGCTGGGGTGGGTTTTCTTTTGGCTGGTGGTGG-3′ (truncated)	13.36 ± 18.62 nM	[63]
	SEB	MB-SELEX	5′-GGTATTGAGGGTCGCATCCACTGGTCGTTGTTGTCTGTTGTCTGTTATGTTGTTTCGTGATGGCTCTAACTCTCCTCT-3′	Nanomolar affinity (exact Kd not reported)	[8]
	BL	GNP-SELEX	5′-CGGGATCCCCGCCGCTGCAGAGGGAGACGCC-3′ (truncated)	17.3 nM	[32]
	V.parahaemolyticus	Cell-SELEX	5′-ATAAGCATGAATTGACCAACCTAAACTTATTCATTTTCCAGCACCTCTAATATTACTGGC-3′	10.3 ± 2.5 nM	[49]
Plant-derived toxin	Ricin	CE-SELEX	5′-ATAGGAGTCACGACGACGACAGAACCGTAGGTTTCGGGCGGAGTTGGTCCGGAAGGTGGCGTGGTATGTGCGTCTACCTCTTGACTAAT-3′	58 nM	[39]
Environmental pollutant	PCBs	FluMag-SELEX	5′-ATACCAGCTTATTCAATTGGCGGGGCTACGAAGTAGTGATTTTTTTCGATGGCGCGTGAGATAGTAAGTGCAATCT-3′	4.02 ± 0.54 uM	[12]
	BPA	GNP-SELEX	5′-CGCCGCCGGAAGAATATAAGGTGCCGTCGCCGCCGCCGC-3′ (truncated)	37.9 nM	[32]

## Data Availability

No new data were created or analyzed in this study.
